# A Frameshift Mutation in the Methyltransferase *rlmN* Is Associated with Increased Linezolid Resistance in *Mycobacterium tuberculosis*

**DOI:** 10.34133/csbj.0090

**Published:** 2026-05-11

**Authors:** Bryn Marie Reimer, Anna G. Green

**Affiliations:** Manning College of Information & Computer Sciences, University of Massachusetts Amherst, Amherst, MA 01003, USA.

## Abstract

**Background:** Linezolid is a key component of treatment regimens for multidrug-resistant and extensively drug-resistant tuberculosis, which is caused by the pathogen *Mycobacterium tuberculosis* (MTB). Resistance to linezolid in MTB has traditionally been attributed to mutations in the 23S ribosomal RNA (*rrl*) and ribosomal protein L3 (*rplC*), but only a fraction of clinically observed linezolid resistance is explained by mutations in these 2 genes. **Results:** We report that an analysis of strains with paired whole-genome sequencing and linezolid minimum inhibitory concentration phenotyping from the Bacterial and Viral Bioinformatics Resource Center reveals that a relatively common frameshift mutation in MTB methyltransferase *rlmN* (5.3% of assessed isolates, encompassing all isolates known to be in the globally distributed MTB lineage 4.10) is significantly associated with increased linezolid minimum inhibitory concentration. In addition to statistical associations, we provide evolutionary evidence of homology to an established linezolid resistance mechanism in *Staphylococcus aureus* and structural evidence that the frameshift mutation likely ablates *rlmN* methyltransferase functionality. **Conclusions:** We find a novel gene associated with increased linezolid resistance in MTB, with potential implications for resistance diagnostics and therapeutic strategies.

## Graphical Abstract

**Figure:**
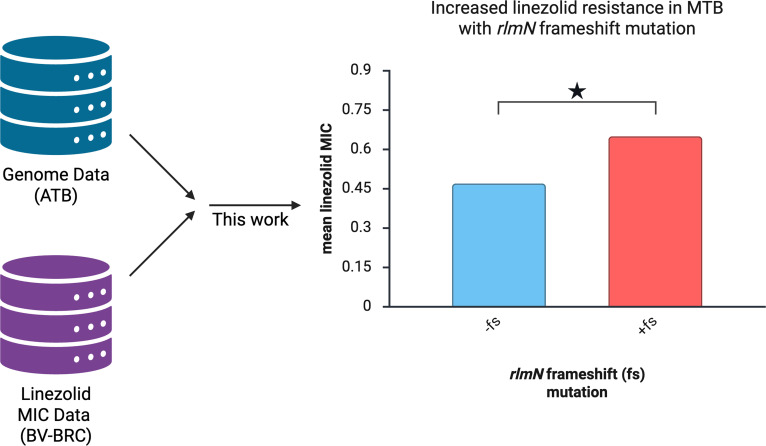

## Introduction

Tuberculosis (TB) remains the world’s most deadly infectious disease, causing an estimated 1.25 million deaths in 2023 alone [1]. TB is caused by the bacillus *Mycobacterium tuberculosis* (MTB), which is uniquely difficult to treat with antimicrobial agents, due in part to its waxy mycolic acid coating [2]. Further complicating treatment is the high rate of drug-resistant infections: in 2023, an estimated 400,000 individuals developed multidrug-resistant or rifampicin-resistant TB, which requires treatment with second-line drugs [1].

Linezolid, a first-generation oxazolidinone, is a commonly used drug in multidrug- and extensively drug-resistant TB [3], and clinical resistance to linezolid is present in an estimated 4.2% of patients with multidrug-resistant TB [4]. The World Health Organization (WHO) maintains a catalog of mutations known to cause resistance to antitubercular drugs, including linezolid, based on stringent statistical criteria applied to mutations in candidate genes [5]. While mutations in *rrl*, the 23S ribosomal ribonucleic acid (rRNA), and *rplC*, ribosomal protein L3, have been observed in linezolid-resistant strains of MTB [6,7], it is unclear what proportion of resistance is explained by these mutations. Estimates vary, but between 20% and 54% of linezolid-resistant isolates appear to have wild-type *rrl* and *rplC* [8,9]. Because a large proportion of clinical linezolid resistance cannot be easily tied to mutations in these 2 major candidate genes, it is reasonable to presume that other genetic mechanisms of resistance may be present.

In this work, we demonstrate that the presence of a frameshift mutation in the ribosomal methyltransferase *rlmN* is associated with increased quantitative linezolid resistance in MTB as measured by minimum inhibitory concentration (MIC). We refer to an association with increased MIC as an association with increased linezolid resistance, because drug resistance as measured by MIC is an inherently quantitative phenotype. The clinical definition of a resistant isolate is one that has an MIC >1.0 mg/l (the WHO breakpoint) [10]; isolates with increased quantitative resistance by our definition may or may not meet the clinical threshold for linezolid resistance. We analyze MTB isolates with paired genotype and phenotype data to show that the presence of this frameshift mutation is significantly associated with increased linezolid resistance. The presence of this frameshift explains 32.1% more clinically resistant isolates compared to using only known resistance-associated mutations from the WHO [5]. Additionally, we contribute a brief analysis of ribonucleic acid (RNA) methyltransferases in MTB to justify our focus on *rlmN*, which is known to cause increased antibiotic resistance in the pathogens *Staphylococcus aureus* and *Escherichia coli* [11,12]. Finally, we contribute a structural analysis of *rlmN* that provides evidence that the observed frameshift mutation would have a deleterious effect.

## Materials and Methods

### Building a catalog of confirmed and putative RNA methyltransferases in MTB

To find all confirmed and putative methyltransferases in MTB, we combined several strategies. First, we searched the standard 2011 H37Rv annotation furnished by Mycobrowser [13] as well as a newly released reannotation of H37Rv [14] for “methyltransferase” and “RNA”. We noted all genes and pseudogenes that were found in this manner (see the Supplementary Materials), whether or not they were included in the final list, and, if not, why.

Additionally, we searched UniProt [15] for methyltransferases thought to have RNA-binding ability. Because some methyltransferases have dual specificity for transfer RNA and rRNA, we searched for any RNA methyltransferase that either was in the methyltransferase class (Enzyme Commission number 2.1.1.*) [16] or was annotated with the Gene Ontology (GO) term for RNA methyltransferase activity (GO:0008173) [17,18]. Specifically, we used the following search terms:

Search 1: “ec:2.1.1.* H37Rv -H37Ra RNA”. We then looked for evidence of ribosomal methylation in the protein annotation.

Search 2: “GO:0008173 H37Rv -H37Ra”.

Any protein that was found by these methods and that seemed likely to have RNA methyltransferase activity based on experimental evidence or homology was included in our catalog.

### Confirmation that no additional *rlmN* homologs are present in MTB

To show that we did not miss any potential *rlmN* homolog(s) in MTB, we performed a tblastn search with the *S. aureus rlmN* protein sequence (UniProt ID: Q2FZ66) against the H37Rv reference genome and confirmed that the known MTB *rlmN* was the only hit produced. Default tblastn parameters were used [19].

### Protein similarity analysis of RNA methyltransferases in MTB

**Fig. 1.**
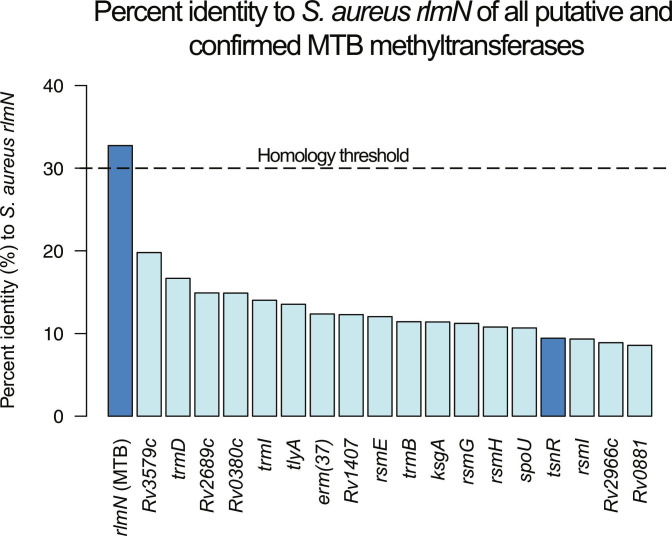
Each putative and confirmed *Mycobacterium tuberculosis* (MTB) methyltransferase is shown with its associated percent identity to the *Staphylococcus aureus rlmN* with 100% query coverage. Highlighted in dark blue are the MTB *rlmN* as well as *tsnR*, which prior literature suggests may be an *rlmN* homolog in MTB. Only the MTB *rlmN* passes the 30% homology threshold to be considered a true homolog.

Protein similarity analysis of the catalog of confirmed and putative RNA methyltransferases in MTB (against the *S. aureus rlmN*) was performed using T-Coffee and European Molecular Biology Laboratory–European Bioinformatics Institute (EMBL-EBI) Job Dispatcher with the default parameters [20,21]. Protein sequences were sourced from UniProt, using the accessions noted in the full text [15]. The percent identity matrix was calculated with T-Coffee [20] and visualized using R version 4.4.2 and the pheatmap library [22,23]. Percent identities shown in Fig. 1 reflect the percent identity over 100% of the query gene length (*S. aureus rlmN*).

### Acquiring MTB isolate phenotype data from the BV-BRC

Information on MTB isolates with lab-based linezolid phenotypes was accessed from the BV-BRC on 2025 July 31 [24]. We downloaded all genome information in the BV-BRC (*n* = 47,497) as well as all phenotype information with “Laboratory Method”-based phenotypes for the antibiotic “linezolid” per the BV-BRC search terms. We then filtered the BV-BRC genomes to only those genomes that contain a lab-based linezolid MIC value (*n* = 9,902), followed by removing duplicate genomes from the list as determined by the BioSample accession values (*n* = 8,439). A detailed Excel file with these calculations is included as a supplementary file.

Most analyses were completed using raw MIC values. For analyses using binarized phenotypes, isolates were labeled resistant if their linezolid MIC was >1.0 mg/l and sensitive otherwise, following the WHO-recommended breakpoint [10].

### Processing uniformly assembled genomes from AllTheBacteria

We used the BioSample accession values from the BV-BRC [24] to query AllTheBacteria [25], a resource supplying uniformly assembled bacterial genomes. All genomes that were available were downloaded (*n* = 8,364). It was noted that a small number of genomes had unusual genome sizes (very large or almost empty) or otherwise poor assemblies. We proceeded to include for analysis only genomes with a size within 5% of the 4,411,532-bp size of the H37Rv reference genome (*n* = 7,775). We confirmed that genomes with aberrant sizes that were excluded were not enriched for resistant isolates (before filtering, 1.71% resistance; after filtering, 1.79% resistance).

We then used BLAST to find our 4 genes of interest in each genome assembly [19]. We used tblastn for the protein sequences (*rplC*, *tsnR*, and *rlmN*) and blastn for the ribosomal DNA sequence (*rrl*). We extracted only the top hit for each search, with an E-value cutoff of 1e–10. After finding all sequences for each genome assembly, we included for downstream analysis only those isolates that had missense, nonsense, or frameshift/indel mutations (*n* = 7,436; 128 resistant, 7,308 susceptible = 1.72% resistance). As before, the proportion of resistant isolates remained similar after filtering. Indels and frameshifts were included only if they were present in greater than 1% of resistant or sensitive isolates, to minimize possible erroneous mutation calls due to the method used for calling mutations. The cutoff was chosen empirically based on ad hoc investigation into the resultant indel/frameshift calls. Because we are using BLAST to search assemblies that comprise some number of contigs, a small fraction of indel/frameshift calls result from the assembly contig ending in the middle of a gene, and therefore, we discarded rare indels and frameshifts. Note that this 1% cutoff is distinct from a variant allele fraction cutoff, as we do not deal directly with raw reads, only with assemblies.

For each mutation detected in each gene, its presence or absence was noted in each isolate. We were then able to calculate the one-sided Mann–Whitney *U* test statistic for each mutation’s association with increased MIC in order to test the null hypothesis that isolates with and without a given mutation are selected from populations with the same MIC distribution. Resultant *P* values were corrected using the Bonferroni procedure. Only mutations significantly associated with an increase in quantitative resistance (MIC) are reported.

### Lineage analysis of select isolates

MTB lineage is typically assessed using lineage-specific single-nucleotide polymorphisms (SNPs) across the MTB genome. Our analysis included only a small subset of genes from the MTB genome, and thus, we could not directly assess the lineage to which isolates in our dataset belonged. However, many of our isolates were derived from the Comprehensive Resistance Prediction for Tuberculosis: an International Consortium (CRyPTIC) data that had been uploaded to the BV-BRC [26]. Their supplementary files include lineage calls for their isolates, where the tool Mykrobe was used, using SNPs from Stucki et al. [27–29].

### Structural insights from *E. coli* RlmN

Protein sequence similarity between *rlmN* in MTB (UniProt ID: P9WH15) and in *E. coli* (UniProt ID: P36979) was performed with BLAST, using default parameters [19]. The protein structure of RlmN was downloaded from the Protein Data Bank (PDB) (PDB ID: 3RFA) [30–32]. The protein–ligand interaction fingerprint (PLIF) was generated using Flare from Cresset and then modified for clarity and color complementarity [33]. Structural modeling was done in PyMOL version 3.1.3 [34].

## Results

### A protein similarity analysis uncovers MTB’s sole *rlmN* homolog

Multiple previous studies have implicated RNA methyltransferase genes in increased quantitative linezolid resistance in other bacterial species. Prior work has proposed a role for the *S. aureus* methyltransferase *rlmN* in creating the correct methylation state of the rRNA to facilitate linezolid binding [11]. In addition, the presence of the plasmid-borne RNA methyltransferase *cfr* in several species such as *S. aureus* and *E. coli* has been associated with linezolid resistance [35]. The importance of these 2 methyltransferase genes in linezolid resistance indicates that the correct methylation state of the rRNA is critical for linezolid function. Therefore, we hypothesize that methyltransferases provide a promising reservoir of genes that may be implicated in linezolid resistance in MTB.

The only prior catalog of all MTB methyltransferases, published in 2016, does not capture some important members of the MTB RNA methyltransferases (see Discussion) [36]. We therefore contribute a systematic catalog of RNA methyltransferases with 2 goals: (a) to provide a candidate gene list for further exploration of drug targets in MTB and (b) to clarify the identity of *rlmN* in MTB, as there has been some confusion in the literature about the identity of *rlmN* in MTB; some have proposed that *tsnR* is its homolog in MTB [37]. Because a loss-of-function mutation in the *S. aureus rlmN* leads to increased quantitative linezolid resistance [11], we hypothesized that if MTB has an *rlmN* homolog, its function may also affect linezolid’s activity. Thus, we proceeded to catalog the RNA methyltransferases in MTB and to align the sequences of these methyltransferases with the *S. aureus rlmN* to find its homolog(s) in MTB (see Materials and Methods).

We identified 19 putative or confirmed RNA methyltransferases in MTB, representing 20 genes in the H37Rv reference genome (Table 1). All 19 genes were found in UniProt, although not all were reviewed entries. The *rlmN* gene as annotated by UniProt is a fusion of Rv2879c and Rv2880c, which are annotated as separate, overlapping genes in the H37Rv reference genome. We confirmed that there were no additional homologs to *S. aureus rlmN* in the MTB genome (see Materials and Methods) that were not recovered by our prior searches through genome annotations.

**Table 1.** A catalog of known and suspected RNA methyltransferases in MTB. Information about gene function (and relevant citations, if any) are from UniProt [15]. A “?” denotes uncertainty about the methyltransferase target(s). * = unreviewed UniProt entry.

Gene identifier	Gene name(s)	UniProt ID	Function/citations
Rv0208c	*trmB*	P9WFY9	(Homology; tRNA)
Rv0380c		O53715*	(Uncharacterized; RNA?)
Rv0881		P9WFY3	(Uncharacterized; tRNA/rRNA?)
Rv1003	*rsmI*	P9WGW7	(Homology; 16S rRNA)
Rv1010	*ksgA*, *rsmA*	P9WH07	(Homology; 16S rRNA)
Rv1407		P9WGX3	(Homology; RNA)
Rv1644	*tsnR*	P94978*	(Uncharacterized; 23S rRNA?)
Rv1694	*tlyA*	P9WJ63	Catalyzes 2′-*O*-methylation at nucleotides C1409 (16S rRNA) and C1920 (23S rRNA); PMID: 40406463, 16857584, 20854656
Rv1988	*erm(37)*	Q10838*	(Uncharacterized; rRNA?)
Rv2118c	*trmI*	P9WFZ1	M1A58 tRNA methyltransferase; PMID: 14960715
Rv2165c	*rsmH*	P9WJP1	(Homology; 16S rRNA)
Rv2372c	*rsmE*	P9WGX1	(Homology; 16S rRNA)
Rv2689c		O07191*	(Uncharacterized; rRNA?)
Rv2879c/Rv2880c	*rlmN*	P9WH15	(Homology; 23S rRNA and tRNA)
Rv2906c	*trmD*	P9WFY7	(Homology; tRNA)
Rv2966c		I6XFS7	Guanine at position 966 of 16S rRNA; PMID: 21474448
Rv3366	*spoU*	O50394*	(Lacks conserved residues; tRNA?)
Rv3579c		P9WFY5	(Uncharacterized; tRNA/rRNA?)
Rv3919c	*rsmG*, *gidB*	P9WGW9	(Homology; 16S rRNA)

MTB, *Mycobacterium tuberculosis*; UniProt ID, UniProt identifier; tRNA, transfer RNA; rRNA, ribosomal RNA; PMID, PubMed identifier

We aligned all putative and confirmed RNA methyltransferase protein sequences in MTB to the *S. aureus rlmN* protein sequence (UniProt ID: Q2FZ66) to find potential MTB homolog(s) (see Fig. 1). Of all putative and confirmed RNA methyltransferases in MTB, the *S. aureus rlmN* is most similar by percent identity (32.73%) to the MTB *rlmN*, whereas it is only 9.43% identical to MTB *tsnR*. It is a common, if conservative, heuristic to assume homology between 2 proteins that have at least 30% sequence identity [38]. Applying this threshold, and noting that BLAST did not find any other potential hits for *rlmN* in the MTB genome, we can confirm that MTB *rlmN* is the sole MTB ortholog to *S. aureus rlmN*. We therefore assert that *tsnR* is not the MTB *rlmN*, nor is it a close homolog of the MTB or *S. aureus rlmN*, despite its similar putative methyltransferase activity and despite the in vitro evidence of *tsnR* association with increased MTB linezolid resistance [39].

### Genotypic analysis recovers known resistance mechanisms and reveals that an *rlmN* frameshift mutation is associated with linezolid resistance

We evaluated the association between genomic variants and linezolid resistance by finding genomic variants in sequenced isolates and assessing their association with a change in the observed MIC for linezolid. By assessing resistance association using MIC values rather than resistance breakpoints, we may be able to find low-level determinants of resistance. After preprocessing to remove low-quality genome assemblies or isolates with missing data (see Materials and Methods), 7,436 isolates were included in our analysis.

We used a candidate gene approach for our genotypic analysis, following an approach used to generate a WHO-endorsed catalog of MTB mutations and whether they cause antibiotic resistance (the “WHO mutation catalog”) [5]. When assessing genotypic variants associated with linezolid resistance, the WHO assesses 3 genes, *rrl*, *rplC*, and *tsnR*, finding mutations significantly associated with resistance in only *rplC* and *rrl* (*rplC* C154R; various in *rrl*). We included these 3 genes and added *rlmN*. We restricted our search to missense and nonsense mutations for proteins *rplC*, *rlmN*, and *tsnR* and to SNPs for the ribosomal genes *rrl*. Indels and frameshifts were included only if they were present in greater than 1% of resistant or sensitive isolates, to minimize possible erroneous mutation calls (see Materials and Methods).

For each mutation found in these genes in our dataset, we used the Mann–Whitney *U* test to test the null hypothesis that isolates with and without a given mutation are selected from populations with the same MIC distribution (see Materials and Methods). We find that 7 mutations are significantly associated with an increase in linezolid MIC in our dataset (see Table 2). No mutations in *tsnR* attained significant association with resistance in our dataset. Of the 7 significantly associated mutations, *rrl* G2814T, *rrl* G2270T, and *rplC* C154R are also associated with resistance in the WHO mutation catalog, which uses binarized phenotypes based on the WHO linezolid breakpoint rather than correlations with an increase in MIC [5]. In addition to these 2 known variants, we also describe 2 other *rrl* variants (C344T and C637G) that are significantly associated with resistance in our dataset but which did not meet criteria for association with resistance in the dataset used by the WHO.

**Table 2.** Observed mutations significantly associated with increased linezolid resistance. This table reports all assessed mutations that are significantly associated with increased linezolid resistance as measured by increased MIC (Bonferroni-corrected *P* values < 0.05). ΔMIC is the change in mean MIC between isolates with and without the mutation. Also shown is the mean MIC for isolates with the relevant mutation (“MIC + mutation (mean)”) and without the mutation (“MIC − mutation (mean)”) as well as the median MIC for isolates with the relevant mutation (“MIC + mutation (median)”) and without the relevant mutation (“MIC − mutation (median)”). All *P* values were calculated using the one-sided Mann–Whitney *U* test and corrected using the Bonferroni procedure. * = this mutation is present only on the background of the frameshift mutation; see the main text.

Gene	Mutation	ΔMIC (mg/l)	MIC + mutation (mean)	MIC − mutation (mean)	MIC + mutation (median)	MIC − mutation (median)	*P* value (corr.)
*rrl*	G2814T	2.37	2.84	0.47	2.0	0.5	4.58e−02
*rrl*	G2270T	2.22	2.69	0.47	2.0	0.5	1.07e−03
*rplC*	C154R	2.05	2.52	0.47	2.0	0.5	2.43e−07
*rlmN*	M176fs	0.18	0.65	0.47	0.5	0.5	1.22e−30
*rrl*	C344T	0.17	0.64	0.47	0.5	0.5	3.33e−19
rlmN*	D244N*	0.17	0.64	0.47	0.5	0.5	3.76e−05
*rrl*	C637G	0.17	0.64	0.47	0.5	0.5	2.06e−05

MIC, minimum inhibitory concentration

**Fig. 2.**
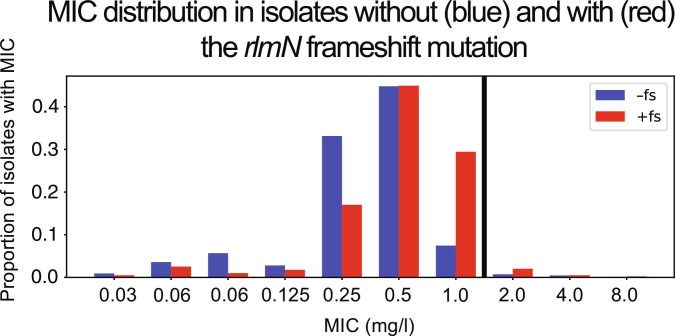
The minimum inhibitory concentration (MIC) distributions are shown for isolates without (blue, −fs) and with (red, +fs) the *rlmN* frameshift mutation. While both distributions have a median MIC of 0.5 mg/l, the +fs distribution shows a rightward distributional shift toward the World Health Organization (WHO) linezolid breakpoint at 1.0 mg/l (shown as a thick black line).

The final 2 observed mutations that are associated with resistance in our dataset are both in *rlmN*. The first is an aspartic acid to asparagine missense mutation at amino acid position 244 in the *rlmN* gene (D244N). The second is a frameshift mutation starting at amino acid position 176. This frameshift mutation is relatively common in our dataset (394/7,436, or approximately 5.3%). When examining raw MIC values, we find that the presence of this frameshift mutation in any isolate is significantly associated with a raw difference of 0.18 mg/l in MIC (Table 2). Visualizing the distributions directly, we observe a right-shifted MIC distribution for isolates with the frameshift mutation compared to those without (Fig. 2), despite the fact that the median MICs remain 0.5 in both cases.

The *rlmN* D244N mutation appears to be significantly associated with resistance in our dataset, but further analysis shows that this mutation is present only on the background of the frameshift mutation. Because D244N mutation appears only downstream of the frameshift mutation at position 176, it is difficult to assess its importance in this dataset. D244N seems likely to be acting as a marker of the frameshift mutation being present.

### The *rlmN* frameshift increases explanatory power for clinical linezolid resistance

The past paradigm in MTB genetics has held that single mutations cause clinical resistance phenotypes, which is known to be true for first-line antibiotics like rifampicin, where single mutations cause large MIC increases that “push” isolate phenotypes above the clinical resistance breakpoints [5]. Because the MIC increase associated with the *rlmN* frameshift is small (0.18 mg/l) compared to the WHO breakpoint for clinical linezolid resistance (1.0 mg/l), we investigated whether the *rlmN* frameshift provides additional explanatory power for designating isolates as clinically resistant.

We binarized the phenotypes using the WHO breakpoint of 1.0 mg/l [10] (isolates with MIC > 1.0 mg/l are resistant) and examined how many resistant isolates harbor the mutations associated with linezolid resistance. Of the 128 resistant isolates in our dataset, only 28 (21.88%) were found to have at least one mutation from the WHO catalog of resistance-associated mutations. When we added just the *rlmN* frameshift mutation to this analysis, 37 (28.91%) isolates harbored the frameshift mutation or at least one mutation from the WHO catalog. Moreover, we observe that the *rlmN* frameshift is more common in linezolid-resistant isolates (11/128, or approximately 8.6%) than in linezolid-susceptible isolates (383/7,308, or approximately 5.2%). Therefore, the frameshift mutation in *rlmN* provides increased explanatory power (32.1% more isolates explained) when assessing the known genetic causes of linezolid resistance.

Our baseline ability to ascribe linezolid resistance to genetic loci is still limited. Of the 128 clinically resistant isolates in our dataset, only 37 (28.91%) have an established genetic cause, which we define as 1 of our 7 reported significantly associated mutations or any WHO-reported linezolid resistance mutation. A past analysis by Kadura et al. sought to explain linezolid resistance by counting whether isolates have any mutations in *rrl* or *rplC*, regardless of whether the mutations are known resistance markers. Across 8 studies with sequencing results for *rrl* and *rplC*, they observed that 85/106 (80.19%) of resistant isolates harbored at least one mutation in those genes [9]. In our dataset, only 42/128 (32.81%) of resistant isolates harbor any mutation in *rrl* or *rplC*. Notably, in our study, 977/7,308 (13.37%) of susceptible isolates still had at least one mutation in *rrl* or *rplC*, but that number drops to 396/7,308 (5.42%) when considering only significantly resistance-associated *rrl* or *rplC* mutations.

### Lineage analysis of isolates in our dataset

We assessed all of our BioSample accessions that were included in analysis (*n* = 7,436) for whether or not they had lineage calls from the CRyPTIC data (see Materials and Methods); 6,217 of our isolates did have lineage calls, meaning 6,217/7,436 = 83.6% of our dataset was covered. Of the 394 isolates with the frameshift mutation, 338 (85.8%) had a lineage call. All isolates with the frameshift mutation were called as lineage 4.10, with the exception of the BioSample with accession SAMEA7525669, which was called as mixed with lineage 4.10 and lineage 4.3.4.1. A total of 339 isolates were in lineage 4.10 or 4.10 mixed, and thus, only one isolate, a BioSample with accession SAMEA7546388, was found to be lineage 4.10 without the frameshift mutation. Only 339/6,217 = 5.5% of isolates in the CRyPTIC data were found to be lineage 4.10, which is consistent with our finding that about 5.3% of assessed isolates in our entire dataset have this frameshift mutation.

### The frameshift mutation will plausibly cause loss of function of MTB *rlmN*

**Fig. 3.**
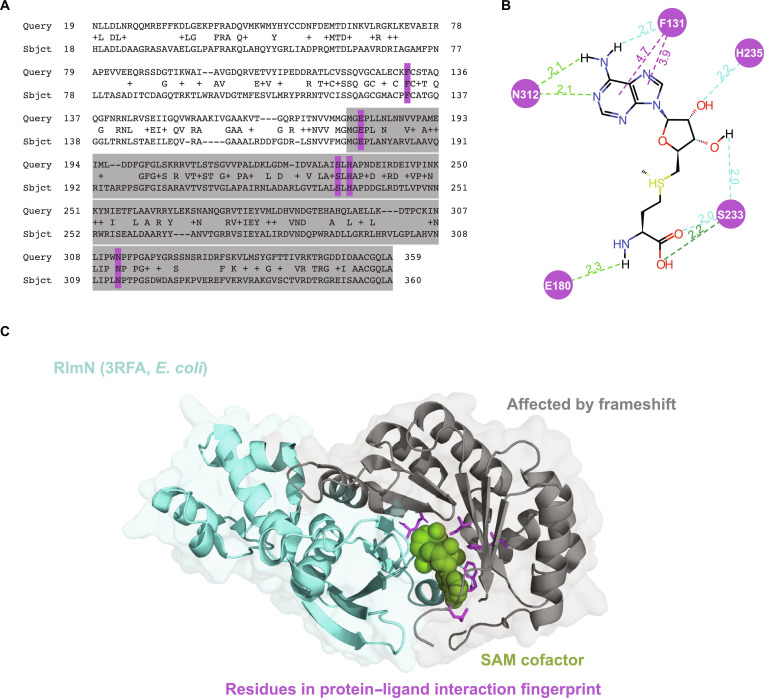
(A) This panel shows the Basic Local Alignment Search Tool (BLAST) alignment of the protein sequences for *rlmN* in both *Escherichia coli* and *Mycobacterium tuberculosis* (MTB), with gray highlights showing the parts of the sequence that would be downstream of the frameshift mutation. Purple highlights indicate important residues in the protein–ligand interaction fingerprint (PLIF). (B) The PLIF, as calculated by the Cresset software (see Materials and Methods), is shown for *E. coli* RlmN (Protein Data Bank [PDB] ID: 3RFA). The green and blue dotted lines indicate hydrogen bonds and weak hydrogen bonds, respectively. The purple lines indicate aromatic interactions. Amino acids are numbered by their index in *E. coli*. Image initially generated using Flare from Cresset and then modified by the authors. (C) The *E. coli* RlmN protein structure is shown, with the area downstream of the frameshift in gray, the *S*-adenosyl methionine (SAM) cofactor in green, and residues important in the PLIF in purple.

To evaluate a possible mechanistic explanation for the observed association, we performed in silico protein structure analyses to assess the likely effect of the frameshift mutation on protein function. While there are no experimentally derived protein structures available for either MTB RlmN or *S. aureus* RlmN, there are several x-ray structures available for *E. coli* RlmN. We aligned the MTB *rlmN* protein sequence with the *E. coli rlmN* protein sequence and found 37% sequence identity across the sequence match, supporting the homology of these 2 proteins (see Fig. 3A). We then generated a PLIF (see Materials and Methods) to assess which amino acids were likely important for binding *S*-adenosyl methionine (SAM), the critical cofactor for RlmN protein methyltransferase activity (see Fig. 3B). The PLIF shows 5 residues with critical binding interactions with SAM, 4 of which are ablated when the frameshift mutation is present. All 5 of these critical binding residues are conserved across *E. coli* and MTB. Although the frameshift mutation occurs only about halfway through the protein, it clearly impacts important residues for cofactor binding and therefore activity (see Fig. 3C), leading us to hypothesize that the frameshift mutation will plausibly cause loss of function of the MTB RlmN. This loss of function, and consequent loss of rRNA methylation, may be the mechanism of the observed association with increased linezolid resistance, and it is consistent with the mechanism of resistance observed in *S. aureus* and *E. coli*, where abolishment of RlmN activity leads to increased quantitative resistance due to the lack of A2503 methylation [11,12].

## Discussion

In this study, we report the existence of a significant association between a frameshift mutation in an MTB methyltransferase, *rlmN*, and phenotypic resistance to linezolid as quantified by MIC. To support our addition of *rlmN* to a small list of candidate genes, we contribute a catalog of all known and putative MTB RNA methyltransferases (Table 1). We demonstrate the homology of MTB *rlmN* to *S. aureus rlmN*, dispelling prior suggestions that *tsnR* may be functioning as an *rlmN* homolog. Upon finding a significant association between a frameshift mutation in *rlmN* and an increase in linezolid MIC, we provide structural evidence for a hypothesized mechanism of resistance, from homology to the *E. coli* RlmN structure.

We also report that we are able to only assign a putative genetic cause of resistance to 37/128 (28.91%) of clinically resistant isolates in our dataset. Thus, there remains a substantial portion of linezolid-resistant MTB isolates where resistance cannot be ascribed to one of the known genetic loci, including our newly discovered *rlmN* frameshift mutation. This phenomenon of unexplained resistance has also been noted in *Mycobacteroides abscessus*, a closely related mycobacterial species that also harbors only one copy of ribosomal DNA [40], where it has been reported that only 8.2% of linezolid-resistant strains contained ribosomal mutations [41]. A recent review proposes that mechanisms involving efflux pumps [41] and the fatty acyl-AMP ligase *fadD32* [42] may contribute to part of the remaining unexplained resistance [37]. Future work assessing the importance of mutations in the MTB homologs of these genes could aid in our understanding of currently unexplained linezolid resistance.

While it may be the case that there are several large determinants of resistance that are missing from our current understanding of the mechanisms of linezolid resistance, it may also be the case that the “missing” resistance is explained by isolates that have gathered several mutations that each confer a small increase in quantitative resistance, and once a certain mutational load is met, clinical resistance (isolate MIC > 1.0 mg/l, the WHO breakpoint) is achieved. Our observed *rlmN* frameshift mutation is one such mutation that is associated with a small increase in MIC, and it may be the case that this mutation, combined with a few other low-level resistance mutations, would “push” an isolate over the clinical breakpoint. We speculate that future genome-wide association-style studies that examine the association of many MTB genetic loci with increased quantitative resistance (right-shifted MIC distribution) might uncover such small-effect-size resistance determinants. This study examined a focused hypothesis generated from the *S. aureus* and *E. coli* literature, but a genome-wide association study would have broader scope.

We also note that in our dataset, this frameshift mutation is present in only a single lineage: lineage 4.10. This lineage comprises around 5% of our dataset and contains isolates that are globally unrestricted, meaning they have broad geographic spread [28]. Lineage 4.10 also contains H37Rv, the typical MTB reference genome. In our study, we treat the UniProt canonical protein sequences as reference for protein comparisons. The strong homology of MTB *rlmN* to other bacterial species’ functional *rlmN* genes and the relative rarity of the frameshift mutation among all samples assessed (5.3%) implies that a working copy of *rlmN* is, in fact, wild-type and this is a case where the MTB reference genome happens to be a mutant compared to the majority of MTB isolates. H37Rv is often considered to be pan-susceptible, and in vitro studies have shown it to have a linezolid MIC of 0.5 mg/l [43]. This finding is consistent with the hypothesis that the *rlmN* frameshift mutation confers low-level resistance that can rise to the level of clinical relevance on a background of other acquired mutations in some strains.

While the *rlmN* frameshift is lineage restricted, we do not believe that it is a neutral mutation; the literature data from *S. aureus* [11] and *E. coli* [12] in vitro studies showing the deleterious effects of *rlmN* mutation, in addition to our understanding of the likely structural consequences of our observed frameshift mutation, lead us to hypothesize the functional consequences of this mutation.

Our hypotheses in this direction are founded upon experimental data in other species as well as our in silico work, but it remains of critical importance that this association between *rlmN* mutation (specifically the frameshift mutation but potentially other *rlmN* mutations as well) and increased linezolid MIC be experimentally validated in a suitable model in order to prove true causality rather than simple association. In vitro work to validate this finding would also improve our ability to understand the clinical relevance of these findings. At present, the clinical relevance of these findings is uncertain: it may be that the *rlmN* frameshift mutation “poises” MTB isolates for clinical resistance (>1.0 mg/l MIC) if more mutations arise on this genetic background, as is suggested by the right-shifting of the MIC distribution curve toward the MIC breakpoint (see Fig. 2). More experimental work would be needed to validate these results and shine a light on the potential clinical applications thereof. Work in *E. coli* shows that the antibiotic pressure of prolonged linezolid treatment on *E. coli* leads to the evolution of antibiotic resistance via *E. coli rlmN* mutation, and these mutations are present in clinical resistance isolates in *E. coli*, which suggests that a clinical impact in MTB may be possible as well, especially if clinical use of linezolid for MTB increases.

Additionally, while the present work focused on a single methyltransferase, *rlmN*, that has compelling evidence for conferring increased linezolid resistance in *S. aureus*, many other MTB RNA methyltransferases may also be involved in creating the correct methylation state for linezolid and other ribosome-targeted therapeutics. There has been growing interest in the plausibility of methyltransferases as drug targets for MTB [44], in no small part due to the importance of MTB methyltransferases such as *tlyA* in capreomycin resistance [45] and *gidB* in streptomycin resistance [46]. We recommend that relevant ribosomal methyltransferase genes be added to candidate gene lists for assessing genotype–phenotype correlations in drug-resistant TB, especially for drugs that target the ribosome. However, we recognize that because the precise mechanism of action is known for only a few of these methyltransferases, picking a targeted candidate gene list based on the proclivity of a gene to methylate near a drug-binding site remains difficult. It is plausible that other RNA modifications, such as formylation, acetylation, or sulfation, may also be important for drug resistance. Knowledge of the precise RNA modifications required for drug activity can help shape structure–activity studies as we continue to develop ribosome-targeted drugs in the future.

To this end, one of our contributions is a systematic catalog of all known or putative MTB RNA methyltransferases. We note that a 2016 review of MTB methyltransferases by Grover et al. [36] suggests the presence of only 16 RNA methyltransferases, in contrast to our 19 identified RNA methyltransferases. Their list of methyltransferases with RNA-binding activity does not recognize the RNA-binding potential of Rv1407, Rv2689c, Rv3919c, and Rv2966c, which our list does due to their homology to known methyltransferases in other species. Rv2966c, in particular, is now an experimentally verified MTB rRNA methyltransferase [47]. Additionally, Grover et al. consider Rv3024c a methyltransferase; however, more recent evidence from 2023 supports the role of Rv3024c as an enzyme that sulfurates (rather than methylates) transfer RNA isoacceptors [48].

In our study, we include 2 methyltransferases as candidate genes for association with linezolid resistance: *rlmN*, our gene of interest, and *tsnR*, which is on the current candidate gene list for linezolid resistance in the WHO mutation catalog [5]. The loss of function of *tsnR* has been observed in vivo to correlate with linezolid resistance [39]. Despite this observation in vitro, no observed *tsnR* mutations met our stringent thresholds for significant association with resistance. This result was also seen in the WHO mutation catalog, which reports no *tsnR* mutations as being associated with resistance.

The association of *rlmN* mutation with linezolid resistance in MTB has not been reported previously. While Li et al. [39] performed a genome-wide CRISPR interference screen that covered 98% of the MTB genome, neither *rlmN* nor its 2 constituent annotated genes in the H37Rv reference genome (Rv2879c and Rv2880c) were targeted by their guide RNAs. Further, the fact that the frameshift mutation appears to be present in the H37Rv reference genome obscures the presence of *rlmN* in MTB by causing difficulty with annotation software; the current Mycobrowser reference genome does not list *rlmN*, and the putative methyltransferase function of the gene is not listed under either Rv2879c or Rv2880c [13]. Recently, however, a comprehensive update to the H37Rv reference genome was released [14]. While the reference genome still contains the *rlmN* frameshift mutation, improved annotation software has now recognized the homology of the gene fragment to *rlmN*. We propose that the community consider adopting this updated 2022 H37Rv reference genome in preference to the 1998 H37Rv reference genome with annotations from 2011 that is currently in use.

In conclusion, we report the novel association between a frameshift mutation in the methyltransferase *rlmN* and linezolid resistance in MTB, as measured by quantitative MIC. This statistical association is observed in a set of 7,436 linezolid MIC phenotyped isolates. Further, we use protein structural studies to hypothesize a plausible causal mechanism of resistance from loss of function due to the ablation of the majority of the critical SAM cofactor binding sites.

As a part of this study, we provide 3 other items of broad usefulness to the scientific community: (a) a catalog of all known MTB methyltransferases, to be used as candidate genes for future drug resistance work for ribosomally targeted drugs; (b) a proposal that *rlmN* be considered as a candidate gene in future WHO mutation catalogs; and (c) an evidence-based recommendation for use of the new, 2022 H37Rv MTB reference genome, which benefits from updated annotations.

Our findings, which unveil a potential novel genetic mechanism underlying linezolid resistance in MTB, have future implications for improved resistance diagnostics and better-targeted therapeutic strategies. Future work experimentally verifying this association is critical to building a better understanding of the role of *rlmN* in linezolid mechanisms of resistance.

## Data Availability

Data supporting the conclusions of this article are available in the GitHub repository, https://github.com/BrynMarieR/rlmN_frameshift. Some data are also included within the article and its supplementary materials. The other datasets mentioned in, but not created for, this study are available from their respective, publicly available repositories as mentioned in the main text.

## Supplementary Materials

Supplementary 1 Data S1. BVBRC linezolid phenotype Data S2. RlmN CRyPTIC reuse table Data S3. rlmN supplementary file
